# A Marine Anticancer Cinnamyloxyl Derivative with Unique Binding Sites at Carbonic Anhydrase IX (CAIX) Inhibits Adenocarcinomic A549 Cells

**DOI:** 10.3390/ph19010132

**Published:** 2026-01-12

**Authors:** Shailaja Vommi Lakshmipathy, Christina Vijayaraghavan Sathyanathan, Mohanapriya Dandapani Chinambedu, Mohanraj Gopikrishnan, Abhinand Ponneri Adithavarman, Sadras Panchatcharam Thyagarajan, Mary Elizabeth Gnanambal Krishnan

**Affiliations:** 1 Department of Biotechnology, Faculty of Biomedical Sciences and Technology (FBMS&T), Sri Ramachandra Institute of Higher Education and Research (SRIHER), Deemed to be University (DU), Porur, Chennai 600116, Tamil Nadu, India; 2 Department of Human Genetics, Faculty of Biomedical Sciences and Technology (FBMS&T), Sri Ramachandra Institute of Higher Education and Research (SRIHER), Deemed to be University (DU), Porur, Chennai 600116, Tamil Nadu, India; 3 Department of Integrative Biology, School of Biosciences and Technology, Vellore Institute of Technology, Vellore 632014, Tamil Nadu, India; 4 Department of Bioinformatics, Sri Ramachandra Institute of Higher Education and Research (SRIHER), Deemed to be University (DU), Porur, Chennai 600116, Tamil Nadu, India; 5 Vellore Institute of Technology (VIT), Vellore Campus, Tiruvalam Rd, Katpadi, Vellore 632014, Tamil Nadu, India; profspt@gmail.com

**Keywords:** carbonic anhydrase IX (CAIX inhibitor), apoptosis, extracellular pH (ΔpHe), anticancer, marine compound, *Cymodocea serrulata*

## Abstract

**Background:** Many inhibitors have been discovered to target hypoxia-induced carbonic anhydrase IX (CAIX) due to its critical role in lung cancers. This study discovers a novel compound, 3-(E-3,4-dihydroxycinnamaoyloxyl)-2-hydroxypropyl-9Z,12Z-octadeca-9,12-dienoate, which is produced by the seagrass *Cymodocea serrulata* and has binding sites at CAIX that are distinct from those of current inhibitors. **Methods:** Compound and reference drug treatment for cell lines; **Cell viability:** MTT; **Staining**: Ao/PI/DAPI; **MMP shifts and cell cycle**: FACS; **Gene and protein expression** of CAIX, *BAX, BAD*: qPCR and Western blotting. **Results:** The compound binds to the CAIX protein, raises extracellular pH, and kills A549 cells [IC_50_: 11.61 µM], producing results that are lower than those of the reference drug doxorubicin [13.7 µM]. The substance depolarised the electrical potential of the mitochondrial membrane, caused S-phase arrest, and fragmented DNA. Additionally, it downregulated CAIX by 0.9 times while increasing apoptotic mRNA, *BAX* and *BAD* by 5.2 and 3.08 times, respectively, as demonstrated by qPCR. Between 0 and 24 h, the untreated hypoxic cells had a ΔpHe of 0.15, but the compound-treated cells had a ΔpHe of 0.6 indicative of intracellular acidosis. MD simulations verify the stability of the CAIX–C1 complex for more than 100 ns, and in silico studies show a strong binding affinity of the molecule to CAIX [−7.55 kcal/mol]. **Conclusions:** This implies that the amount of extracellular alkalosis was increased by the combination of treatment and hypoxia induction. As a result, when the cells were deprived of O_2_, the compound provided less defense against ROS. The compound binds to the glutamine and alanine amino acids at positions 242 and 392, respectively, at the central Zn atom of CAIX, which sets it apart from conventional sulphonamide CAIX inhibitors. This naturally occurring compound may be a potent CAIX inhibitor with newer binding sites, which could help treat hypoxic lung cancers.

## 1. Introduction

Rapid cell division in tumours causes a disruption of the surface and flow in the microcirculation, ultimately leading to tissue hypoxia [1]. This occurs in the activation of the cellular response, hypoxia-inducible activator of transcription factor-1α (HIF-1α). The accumulation of HIF-1α leads to the transcription of downstream factors including vascular endothelial growth factor, erythropoietin, glycolytic enzymes, and carbonic anhydrase IX (CAIX) [2]. Carbonic anhydrase is a family of metalloenzymes that play a role in the regulation of intracellular hormones and cytokines as well as in numerous tumours. Sixteen isoforms have been identified which show different subcellular localisation, catalytic activity and tissue distribution [3]. CAIX has been shown to be overexpressed in many cancers. Since the identification of CAIX (later known as the G250 antigen) in 1986, many studies have appeared in the scientific literature regarding its value as a diagnostic tool, biomarker, and therapeutic target [4].

CAIX, a member of the α-carbonic anhydrase family, is well known to be associated with tumours, as it is induced in response to hypoxia. CAIX is a zinc metalloenzyme which catalyses carbon dioxide to bicarbonate ions and protons, thereby leading to the survival of cancer cells in hypoxic conditions [5]. It is commonly recognised that CAIX is essential for lung tumours in both A549 cells and non-small cell lung cancers (NSCLCs), and multiple research studies suggest this fact [6,7]. Rapidly multiplying cancer cells have more energy requirements than normal cells of the same cell type. Therefore, excessive glucose usage builds up as lactate, which causes intracellular acidosis sufficient enough to damage the growing cells. Thus, CAIX is important in driving acid-producing species out of the cells, thereby reducing intracellular acidosis. It is a known fact that hypoxia induces the overexpression of CAIXs, in particular, lung [8] and cervical and pancreatic cancer morphotypes [9]. In this regard, newer CAIX inhibitors are constantly explored because existing CAIX inhibitors lack specificities owing to homologies in carbonic anhydrase (CA) isoforms.

Numerous results support the notion that CAIX plays a crucial role in lung malignancies, including NSCLCs and A549 cells [6,7,10]. Furthermore, it was demonstrated that hypoxia causes CAIXs to be overexpressed compared to normoxic settings, specifically in lung malignancies over cervical, pancreatic, and other morphotypes [9].

Intending to target CAIX in aggressively growing A549 cancers, the present study explores the anticancer potential of an organic compound [IUPAC name: 3-(E-3, 4-dihydroxycinnamaoyloxyl)-2-hydroxypropyl 9Z, 12Z-octadeca-9, 12-dienoate]. This compound (called C1, henceforth) is isolated from the seagrass *Cymodocea serrulata*. Prior research in our lab revealed that C1 had an IC_50_ value of 5.8 µM for killing human ovarian cancer cells, PA1 [11]. Incidentally, the compound also had an inhibitory effect in A549 alveolar epithelial cell lines at an IC_50_: 11.61 µM, possibly by inhibiting the enzyme CAIX, which was predicted as a potent target in silico. It was found that the compound caused DNA fragmentation, cell cycle arrest and dysregulated mitochondrial membrane potential (MMP) in the human lung cancer cell line A549. A quantitative determination of *CAIX* mRNA and the genes responsible for cell survival and apoptosis was made using real-time PCR. It was discovered that the compound induced apoptotic mRNA transcripts and inhibited and downregulated *CAIX* to cause cell death. Additionally, the compound-treated cells expressed less CAIX, according to Western blotting. Extracellular pH (pHe) and lactate levels were determined in the spent medium of the treated and untreated cells in order to establish a connection with CAIX protein expression. Molecular modeling indicates that C1 interacts with the Zn-binding region of CAIX differently with the widely used sulphonamide/coumarin inhibitors. Therefore, this study identifies distinct binding sites at CAIX, demonstrating the usefulness of naturally occurring marine compounds as CAIX inhibitors to address the present lack of selectivity of pharmacological inhibitors to CA isoforms.

## 2. Results

### 2.1. C1 Compound Caused DNA Fragmentation, Cell Migration Inhibition, S Phase Arrest, and Depolarised Mitochondrial Membrane Potential (ΔΨm) and Upregulated Apoptotic mRNAs

**Figure 1:**
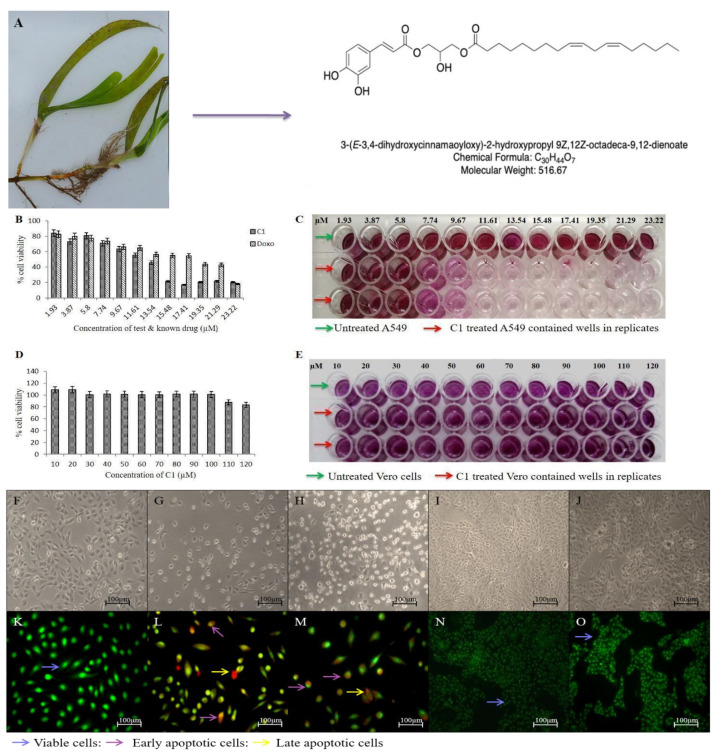
Captured image of *Cymodocea serrulata* during field collection and the chemical compound (C1) used for the study (**A**). Assessment of cell viability of cancerous A549 (**B**) and non-cancerous Vero cell lines (kidney epithelial cells isolated from African green monkey) (**D**) per MTT assay and the corresponding assay plates for reference (**C**,**E**). Panels (**F**–**J**) indicate A549 cells untreated, C1-treated, Doxo-treated, Vero cell line untreated and C1-treated, respectively, in phase contrast mode. The same is applicable to the panels (**K**–**O**) but stained with acridine orange–propidium iodide counter-stained (AO/PI) in the same order. Note: 1 µg/mL concentration of C1 corresponds to 1.93 µM.

**Figure 2:**
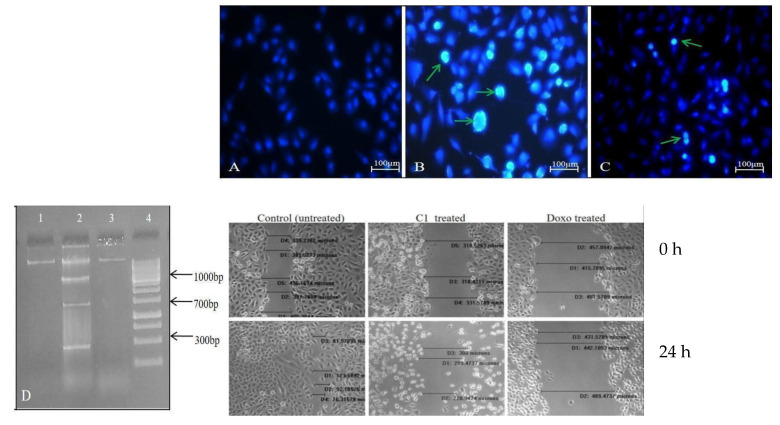
Control (**A**), C1 (**B**) and Doxo-treated (**C**) A549 cells stained with DAPI to indicate intact or condensed DNA (as green arrows) and DNA laddering as (**D**). Cell migration assay performed at 0 and 24 h: C1 as well as Doxo-treated A549 cells showed hindrance to migrate.

**Figure 3:**
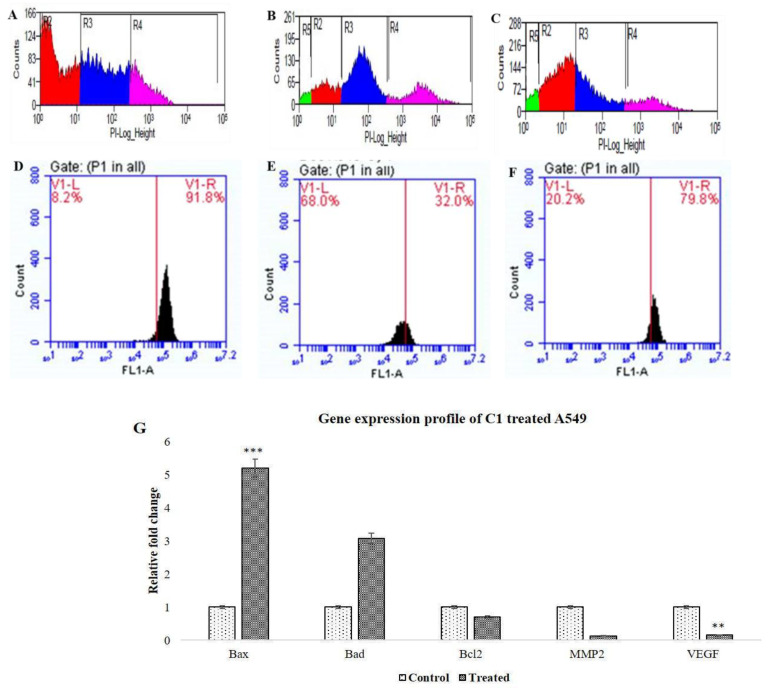
FACS data indicate the distribution of untreated A549 cells in all phases of cell cycle (**A**) and at S phase (**B**,**C**) upon treatment with C1 and Doxo, respectively. Mitophagy-induced cell death is shown as disturbed MMP signals with reduced Rhodamine-123 fluorescence for the treated (**E**) and Doxo (**F**) as against controls (**D**). Upregulated apoptotic mRNAs vs. concomitant reduced levels of cell survival mRNAs with emphasis on *BAX* points to the possibility of intrinsic apoptosis (**G**) [statistically significant differences ** at *p* < 0.05 and *** at *p* < 0.005].

A549 cells were killed by C1 and Doxorubicin (Doxo) in a dose-dependent manner with IC_50_ values of 11.61 µM and 13.7 µM, respectively. C1-introduced cells had a reduced cell count and a distorted morphology (spherical-shaped cells). Additionally, distinct cellular and nuclear alterations were seen, such as nuclear and chromatin condensation and nuclear blebbing, which were not present in the untreated or non-cancerous Vero cell lines. While there was no cell death in the non-cancerous cell lines, A549 cells stimulated by C1 displayed a mixture of AO/PI-stained populations comprising early apoptotic cells (greenish orange) and late apoptotic cells (red). Even at concentrations ≈10 times (120 µM) greater than the IC_50_, Vero cells exposed to C1 showed no cell death, demonstrating its non-toxic nature towards normal cells. (Figure 1). A549 cells treated with C1/Doxo showed fragmented genomic DNA, and both treatment groups showed impaired cell motility. As seen in Figure 2, C1-introduced A549 cells barely invaded 14.47 ± 8.4% of the gap, but the untreated cells covered up the scratched area to 78.37 ± 3.72% (* *p* < 0.0129). In contrast to unstimulated cells (28.89 ± 5.1%), C1-stimulated cells had a 1.8-fold increase in cell population in the S phase (46.49 ± 3.4%), suggesting cell arrest in the synthesis phase. Additionally, a significant portion of cells accumulated in the sub G0 phase (3.88 ± 0.29%), which is indicative of apoptosis. Furthermore, 68% of cells treated with C1 exhibited dispersed Rhodamine 123 fluorescence, confirming depolarised ΔΨm and intrinsic apoptosis. Apoptotic mRNAs were clearly upregulated, as evidenced by a 5.2-fold change in *BAX* (** *p* value < 0.009) and a 3.08-fold change in *BAD* (*p value < 0.07), with a corresponding downregulation of cell survival genes (0.7 times) for *BCL2* (*p*-value < 0.7). Additionally, there was a 0.8-fold reduction in MMP2 and VEGF gene expression, which might have prevented cell migration. Figure 3A–G shows the FACS measurements and mRNA expression levels of the gene panels employed in the experiments.

### 2.2. Docking Analysis

**Figure 4:**
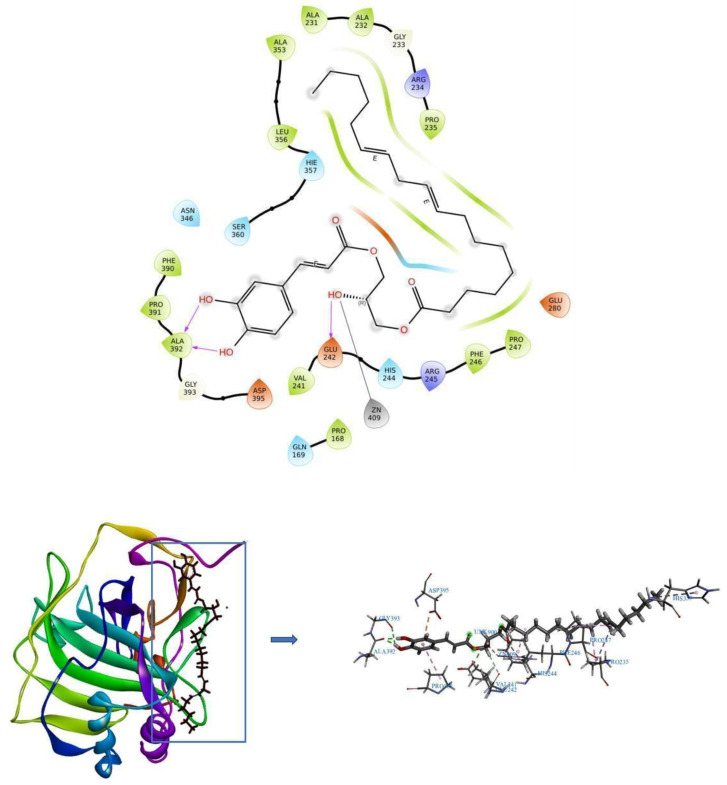
The 2D (**up**) and 3D (**down**) interactions of C1 (crimson red) with CAIX at GLU242 and ALA392 at the Zn coordinate, different from the existing CAIX inhibitors.

CAIX is highly expressed in the tumour microenvironment in response to hypoxia [12]. This is perhaps because vigorously growing tumour cells lack oxygen and thus start expressing Hypoxia-Inducible Factor 1 (HIF-1) for survival. HIF-1 production promotes the expression of various proteins that support the growth of cancer cells in hypoxic conditions [13], and one of the most predominant is CAIX. It is a membrane-bound enzyme that catalyses the formation of protons and bicarbonate ions from carbon dioxide and drives them out of the cell to prevent intracellular acidosis [14]. Protein–protein interactions clearly show associations between HIF-1A and CAIX, indicating that these two proteins function closely with each other. For performing molecular docking between CAIX and the compound C1, the 3D structure of CAIX was obtained from the Protein Data Bank with the corresponding PDB ID being 5DVX. The binding affinity of protein–ligand complexes was evaluated using docking analysis. Greater binding energy values indicate more potent inhibitory effects and enduring complexes. The binding affinity of CAIX and C1 complexes was −7.5 kcal/mol. This interaction included the creation of three hydrogen bonds with the residues GLU242 and ALA392 playing a role in the interaction. Table 1 summarises the binding affinities, the count of hydrogen bonds, and the associated interaction residues. Figure 4 depicts the 2D and 3D interactions of apo CAIX and CAIX–C1, respectively.

### 2.3. Molecular Dynamic (MD) Simulation

MD simulations were conducted to evaluate the interaction mechanisms and stability of potential ligands within human carbonic anhydrase IX complexes, including both the CAIX–C1 complex (with the Gelatinase A-PEX inhibitor) and the unbound CAIX protein in water. An evaluation was conducted on the docked complexes, including the (Root Mean Square Deviation (RMSD), Radius of Gyration (Rg), Root Mean Square Fluctuation (RMSF), Solvent Accessible Surface Area (SASA), potential binding energy, hydrogen bond analysis, Principal Component Analysis (PCA), and Free Energy Landscape (FEL).

#### 2.3.1. Root Mean Square Deviation

**Figure 5:**
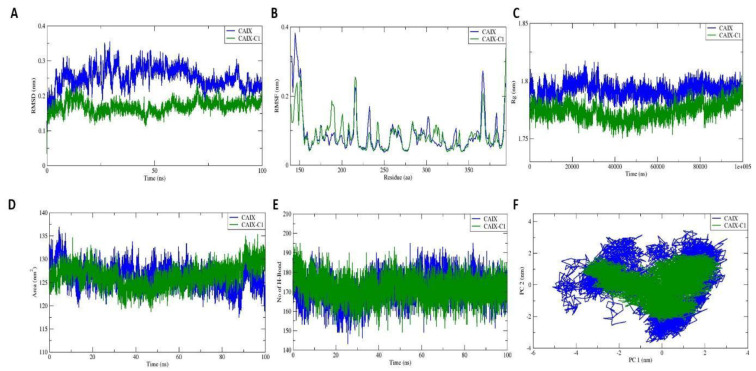
Evaluation of (**A**) root mean square deviation (RMSD), (**B**) root mean square fluctuation (RMSF), (**C**) radius of gyration (Rg), (**D**) solvent accessible surface area (SASA), (**E**) intra-hydrogen bond formation, (**F**) principal component analysis plots during 100 ns in molecular dynamic simulations. The apo protein CAIX is represented in blue, while the complex of CAIX protein with C1 ligands is represented in green.

The duration for the simulated system to attain structural equilibrium was ascertained through the calculation of RMSD, which is a crucial metric for assessing variability or alterations in the molecular structure of a protein. The equilibrium time for both CAIX with C1 complexes and the apoprotein was estimated through the backbone RMSD during a 100 ns MD simulation, as illustrated in Figure 5A, which displays the RMSD values of protein backbones. In the apo CAIX protein, an initial slight fluctuation is observed until 10 ns; then, it reaches stable equilibration with the average RMSD of 0.30 ± 0.04 nm. However, the ligand complexes of CAIX–C1 exhibit lower RMSD values than the apoprotein with an average RMSD of 0.14 ± 0.01 nm, respectively. Larger RMSD values imply reduced stability, while smaller RMSD values suggest more stability within the complex. This suggests that the binding of the ligand contributes to the overall stability of the CAIX–C1 complex.

#### 2.3.2. Root Mean Square Fluctuation

The RMSF parameter, measuring residue-induced stability fluctuations in a complex structure, revealed slight fluctuations in the protein’s backbone. The average RMSF value was 0.10 ± 0.09 nm for the apoprotein and 0.08 ± 0.04 nm for the ligand complexes, as illustrated in Figure 5B. These results suggest that the ligand complexes exhibit a slightly lower degree of fluctuation, indicating potential increased stability compared to the apoprotein. Higher fluctuation levels are associated with destabilisation of the protein structure, emphasising the stabilising influences of ligand binding in the CAIX–C1 complex.

#### 2.3.3. Radius of Gyration

Determining Rg involved measuring the protein’s molecular volume and density, providing insights into its structural characteristics. Rg, defined as the root mean square distance between every atom in the system and its centre of mass, served as a crucial metric. The protein–ligand complexes exhibited consistent Rg values measuring 1.75 nm for the ligand complex and 1.80 for the apoprotein, as illustrated in Figure 5C. The consistently low Rg values observed throughout the 100 ns simulation suggest that the protein maintains a compact structure. Importantly, this implies the stability of inhibitory binding, contributing to the overall structural integrity of the system.

#### 2.3.4. Solvent Accessible Surface Area

The SASA was computed to evaluate simulated system stability and quantify solvent exposure to the receptor region. A higher SASA value indicates increased protein volume throughout the MD simulation. The ligand’s interaction at the active site can affect the protein’s folding and surface area (SASA). As shown in Figure 5D, the average SASA value for the apo CAIX protein and ligand complexes was 126.24 ± 2.47 nm^2^ and 123.96 ± 2.06 nm^2^, respectively. These values imply that the protein structure remained largely unaffected by the binding process. Importantly, it suggests that the complexes maintained stability following the binding of ligands within the active region. In summary, the SASA reinforces the stability of the ligand with bound complexes and their minimal impact on the overall structure of the CAIX protein.

#### 2.3.5. Hydrogen Bond Formation

The intra-bond analysis assessed potential structural interferences regarding hydrogen bonding within the proteins. The apo CAIX protein and ligand CAIX–C1 complexes exhibited 173 and 170 hydrogen bonds, respectively, as represented in Figure 5E. This observed trend in hydrogen bond formation may contribute to the stability observed in RMSD, Rg, and SASA values. The increased number of intra-hydrogen bonds plays a stabilising role in the ligand-docked complexes.

#### 2.3.6. Principal Component Analysis

The overall atomic movements were analysed using the main components, PC1 and PC2. PC1 captures the primary direction of variations, whereas PC2 captures the secondary direction of variations. The eigenvector determines the movement of the Cα atom in the CAIX protein when it is bound to the ligand complex. The CAIX proteins and their corresponding ligand complexes are graphically depicted in Figure 5F. The graph depicts a 2D projection of the trajectory, featuring the projection of eigenvector 1 projected onto the X-axis and eigenvector 2 along the Y-axis. The co-localisation of protein and ligand complexes suggests a greater likelihood of increased stability. On the other hand, it takes up a significant amount of space, which suggests less stability throughout the interaction in the dynamics. The ligand-docked CAIX–C1 complex explores a smaller range of conformational space and is more stable than the apo CAIX protein. The apoprotein has shown a greater range of conformations in the PCA analysis, resulting in decreased stability.

#### 2.3.7. Free Energy Landscape

**Figure 6:**
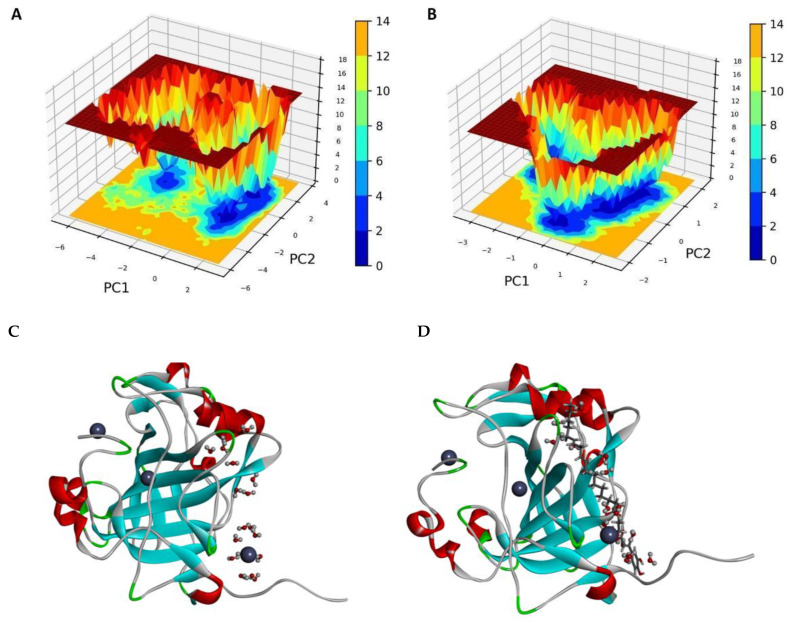
Free energy landscape of (**A**) apo CAIX protein and (**B**) CAIX with C1 complexes, respectively Warmer regions (red, yellow, white) typically indicate areas of high free energy corresponding to energy barriers, whereas, cooler colors (e.g., dark blue, black, green) typically indicate regions of low free energy and these regions correspond to stable or highly probable conformations of the molecule (e.g., the native, folded state of a protein) where the system is likely to reside for longer periods. We can understand there is a considerable area of low free energy states that can facilitate stable conformations both for native CAIX as well as C1-bound forms.3D representation of protein with ligand complexes along with water after 100 ns MD simulation. (**C**) apo CAIX protein, (**D**) CAIX with C1 complexes, respectively.

To investigate structural changes resulting from ligand binding in CAIX, FEL was constructed for both CAIX and CAIX–C1 ligand complexes. The FEL plots were generated using PCA’s top two principal components (PC1 and PC2). The purple and deep blue areas in the FEL basin represent states with low energy or native conformations. In contrast, orange and deep red areas correspond to states with high energy or unstable conformations. The CAIX–C1 complex displays a global energy minima basin, indicating a stable conformational state. In contrast, the apo CAIX protein exhibits multiple transition states and two global energy minima, signifying lower stability than the CAIX–C1 complexes, as illustrated in Figure 6. This figure shows a 3D representation of protein with ligand complexes and water after 100 ns MD simulation, with apo CAIX protein (Figure 6C) and CAIX with C1 complexes (Figure 6D).

#### 2.3.8. CAIX Downregulation Coincided with Elevated Extracellular pH (pHe) and LDHe in Both Normoxia and Much Higher in Hypoxic A549 Cells

It was known that cells treated with C1 had 0.9-fold lower levels of *CAIX* mRNA. Extracellular pH (pHe) and lactate dehydrogenase (LDHe) were evaluated to determine whether or not C1 also affects *CAIX* function. In order to determine if hypoxia-induced C1-treated cells exhibited lower pHe levels than normal cells due to decreased *CAIX* expression, studies were conducted in two groups: normoxic conditions and hypoxic conditions. This is because it is known that hypoxic conditions raise *CAIX* levels. Because CAIX also interferes with lactate release in tumour microenvironments, LDHe levels were also tracked. When compared to the untreated controls (ΔpHe: 0.15), the C1 (ΔpHe: 0.3) and Doxo-treated (ΔpHe: 0.4) groups had two and three times less extracellular acidification between 0 and 24 h, respectively. This demonstrates that C1 therapy significantly decreased extracellular acidosis, leading to an increase in intracellular acid buildup that can be linked to normoxic CAIX inhibition.

**Figure 7:**
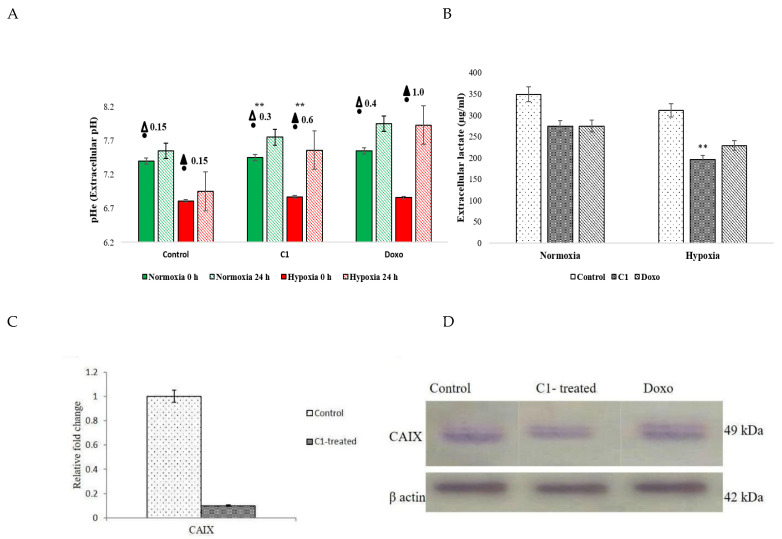
Increased pHe in treatments: normoxia (green) and hypoxia (red) [Solid fill: 1 h; lines: 24 h]. Concomitant reduction in LDHe (**A**) in C1 (black pattern) and Doxo-treated (Grey pattern) than untreated controls (white pattern [** statistically significant differences at *p* < 0.05 and 0.005, respectively [Student’s *t*-test]). Relative difference in pH of normoxic (ΔpHe) and hypoxic conditions (▲pHe) between untreated and treated cells. LDHe reduction in C1-treated cells (**B**). Decreased mRNA (**C**) and protein expression of CAIX in treatments (**D**).

However, whether treated with C1 or Doxo, hypoxia induction significantly reduced extracellular acidosis more than normoxic cells. For instance, when the pHe difference (ΨpHe) for the untreated hypoxic cells was 0.15, it was 0.6 for C1-treated cells and 1 for Doxo-treated cells (as shown in Figure 7). This indicates that in hypoxic conditions, CAIX can increase pHe differences to a statistically significant level if it is downregulated or not functional, leading to a significant surge in cellular acidosis. Likewise, LDHe levels in normoxic cells treated with either C1/Doxo were found to be lower than the control, at 273 ± 1.9 µg/mL and 274 ± 1.3 µg/mL, respectively. This suggests that the treated groups were less protected against oxygen-linked stress. In summary, C1- and Doxo-induced normoxic and hypoxic-grouped A549 cells had increased intracellular acidification alongside LDHe levels. A brief overall comparative analyses of certain properties of C1 and Doxorubicin are indicated in Table 2 for better understanding.

## 3. Discussion

One could argue that the esterase capabilities of many alpha CAs naturally could break down the compound C1, which is an ester of cinnamic and linoleic acids [15]. Fortunately, glycerol shields the ester activity of C1 and in fact, glycerol esters are protected from CAs’ esterase action. The current molecule does not display esterase activity, unlike glycerol esters with shorter acyl chain lengths, such as those with less than ten carbon atoms. Glycerol esters are essentially shielded from esterases by enzyme specificity. As a result, C1 is less vulnerable to inherent esterase activity of the alpha CAs like CAIX.

The results of the current study make it abundantly evident that C1 was safe for non-cancerous cells and effective against epithelial A549 lung cancer cells without harming non-cancerous Vero cells. In our earlier study, it not only inhibited A549 but also human ovarian tumor cell lines, such as PA1 [IC_50_: 5.8 µM] [11]. Specifically, C1 caused DNA condensation and fragmentation to kill A549 cells with an IC_50_ of 11.61 µM. Additionally, cell migration was inhibited; in the flow cytometry plot, 68% of the cells were moved to the MMP-compromised region, demonstrating the function of the natural compound as an intrinsic apoptosis-inducing agent. Within 24 h of therapy, the pro-apoptotic genes *BAX* and *BAD* markedly went up by three and five orders of magnitude, respectively, whereas the cell survival genes *BCL2*, *MMP2*, and *VEGF* decreased. Real-time apoptotic effector expression of *BAX* and *BAD* mRNA transcripts was frequently accompanied by DNA condensation, fragmentation, and damaged mitochondrial membranes. The disturbed cells experienced an induction of S phase accumulation.

One of the top ten possible targets for C1 was identified in silico as the enzyme CAIX. Zinc metalloenzymes called CAs efficiently contribute to the reversible hydration of carbon dioxide, hence controlling intracellular pH. The role of CAs is well established for malignant cells over normal cells, since maintaining a more likely alkaline pH appears to be favorable in the growth and migration of cancer cells. This explains why CAIX expression is rather high, out of the 15 CAs that have been found so far. CAIX is a transmembrane glycoprotein that is part of the alpha class of CAs. It has a catalytic site that points extracellularly and is known to have the largest proton transfer from the cell to the outside of all CAs, creating an acidic milieu. Tumour development, extracellular matrix degradation, chromosomal rearrangements and growth factors are all strengthened by the continuous extracellular acidification to sustain a robust tumour microenvironment. According to reports, malignant cells of the same kind have consistently higher levels of CAIX mRNA and protein expression than normal cells [16,17]. Therefore, it is commonly accepted that CAIX inhibition causes an alkaline extracellular pH and a significant reduction in intracellular pH, which encourages tumour cells to engage in autophagy [18,19]. The target was predicted in silico using the docking score (−7.55 kcal/mol) that developed between the CAIX–C1 complexes. The molecular dynamics investigations provided strong evidence for this when an apoprotein and the C1-protein complex bound steadily for 100 ns. Together with understanding the conformity of the docked complex over time, fluctuations in the individual amino acids were also monitored using root mean square fluctuations (RMSFs), and there was an average fluctuation of 0.10 ± 0.09 nm for the apoprotein and 0.08 ± 0.04 nm for the ligand complexes [20,21]. It was seen from the type of interaction that C1 formed more frequent ionic bonds with glutamine and alanine amino acids at positions 242 and 392, respectively, when bound to Zn metal. Compound C1, an ester of 3,4-dihydroxycinnamic acid, piles up cells at the S phase, indicating that G0/S transition was permitted, whereas some sulphonamide-based CAIX inhibitors, such as S1 [22], indisulam [23], ureido-substituted benzene sulphonamides (USBs) [24], and the naturally occurring coumarin [25] inhibit transition at the G0/S phase. Since all of the aforementioned compounds bind universally at the zinc-attached conserved histidine residues, 94, 96, and 119, interestingly, C1 binding to the zinc ion at the base of the active site cleft in CAIX is coordinated by GLU242 and ALA392 [Crystal structure: PDB ID: 3IAI].

Zinc ions can attach to aliphatic secondary hydroxyl (–OH) groups, and this interaction is crucial for numerous chemical and biological processes. Electrostatic forces caused zinc, a positively charged metal ion, to bind with the negatively charged oxygen in the hydroxyl group of C1. In living systems, this kind of interaction is essential for zinc transport and enzyme catalysis. Alcohol groups’ oxygen atoms are among the several ligands that zinc ions can pair with. The stability and reactivity of the hydroxyl group and the zinc ion are both impacted by this coordination. In conclusion, biological processes can be significantly impacted by their interaction with the aliphatic secondary –OH group, as in C1, especially the catalytic activity of the central coordinate, Zn-CAIX [26]. Consequently, these amino acids are essential for preserving the integrity of CIAX’s active site. Therefore, C1 may provide opportunities to investigate novel binding locations and, consequently, differences in enzyme inhibitory activity when bound to these essential amino acids.

Real-time mRNA expression and the subsequent protein expression of CAIX were particularly carried out in order to validate the anticipated targets in silico. mRNAs for *CAIX* were found to be 0.8 times lower than those of the control cell population; the amount of protein expression was also somewhat lower. It is well established that CAIX inhibitors lower CAIX expression at both the mRNA and protein levels [27]. The selective expression of CAIX across cell types can account for the findings of the current investigation, which indicate decreased CAIX at mRNA but not at statistically significant levels for protein expression. For instance, whereas an NSCLC, which has a more aggressive phenotype, can conceal the effects of chemical treatment, some cell cancer types may not have oncogenic characteristics high enough to cause increased CAIX expression [12]. Additionally, it has been demonstrated that CAIX diffuses from the tumour cells into the culture media and is thus identified in both the cell’s intracellular and extracellular compartments, resulting in unclear CAIX signals on the blots [28,29].

Extracellular pH and LDH levels for cells stimulated with both C1 and Doxo were monitored as separate groups to determine whether or not C1 can inhibit CAIX. Numerous CAIX-targeting compounds have been shown to increase pHe and decrease LDHe levels in a concentration-dependent manner. These compounds include nitro-imidazoles [30,31], sulphonamides [32], and fluorinated sulphonamide-based [9] inhibitors. Aggressive tumours are known to produce Hypoxia Inducible Factor 1α (HIF-1α), which improves the ability of CAIX to remove acid-producing components from the cell [33]. The hypoxia-driven overexpression of CAIX influences the conversion of carbon dioxide (CO_2_) to protons (H^+^) and bicarbonates (HCO_3_^−^) to maintain an alkaline intracellular pH (pHi) and an acidic extracellular pH (pHe). The results of the current studies show that hypoxia intervention for C1/Doxo therapies both raised extracellular pH (pHe) and concurrently decreased LDHe, especially under hypoxic settings. In hypoxic settings, the difference in extracellular alkalinity between 0 and 24 h was 0.6 for C1-induced and 0.15 for untreated. In contrast, the difference in extracellular alkalinity (ΔpHe) between 0 and 24 h under normoxic circumstances was only 0.3 in the C1-induced group and 0.15 in the untreated group. This highlights the involvement of C1 in providing less protection when the cells were especially challenged with O2, yet it is impossible to ignore the lower risks of normoxia per se. Similar to the earlier findings, C1 significantly reduced LDHe levels when hypoxia was induced (78.59%) compared to when the cells were not challenged (62.7%) with O_2_. These findings unequivocally show that under normal circumstances, C1 raised extracellular pH and decreased LDHe. It can be used to specifically inhibit CAIX in lung cancer genotypes that develop more aggressively in oxygen-free environments since the effects were more noticeable in hypoxic circumstances.

## 4. Conclusions

An integrative set of A549 cell-inhibitory effects of 3-(E-3, 4-dihydroxycinnamaoyloxyl)-2-hydroxypropyl 9Z, 12Z-octadeca-9, 12-dienoate isolated from a seagrass, *Cymodocea serrulata,* is presented in this paper. The effects included hindrance to cell movements, DNA fragmentation, S phase arrest, reduction in ΔΨm, changes in genetic profiles and the real-time expression of pro- and anti-apoptotic genes. The gene and protein expression of CAIX cells treated with C1 reveals a downregulation, which was well correlated with increased pHe and reduced LDHe levels. The effect was more pronounced for the hypoxic A549 cells. The molecular docking score for the CAIX–C1 complex was favourable (−7.55 kcal/mol), and the binding was maintained for over 100 ns without any observable loss in the conformity for the bound protein when compared with apoprotein. C1 formed most frequently ionic bonds with glutamine and alanine amino acids at positions 242 and 392, respectively, attached to Zn metal, as seen from the type of interactions, which was unlike the binding sites (at positions 94, 96, and 116) with which known CAIX inhibitors usually interact. Therefore, more specificities for CAIX, especially for better inhibition, may be possible with the discovery of new binding sites for the Zn-interacting CAIX inhibitor. This work presents a broad opportunity to investigate C1 for CAIX inhibition (which binds to more recent locations in CAIX than the current CA inhibitors) for a variety of malignancies that thrive in oxygen-free environments.

## 5. Materials and Methods

### 5.1. Materials

Culture media, stains, buffers and media supplements which are routinely used for in vitro studies [Dulbecco’s Modified Eagle Medium (DMEM), Trypsin-EDTA, antibiotics-antimycotics, 3-(4, 5-dimethylthiazol-2yl)-2, 5 diphenyl tetrazolium bromide (MTT), acridine orange (AO), propidium iodide (PI), 4, 6-diamidino-2-phenylindole (DAPI)] and cobalt chloride were purchased from HiMedia (Mumbai, India). Fetal Bovine Serum (FBS) from Gibco (New York, USA), dimethyl sulfoxide (DMSO) from HiMedia (Mumbai, India), fine grade agarose from Lonza (Basel, Switzerland) and Rhodamine 123 and RIPA buffer were acquired from Sigma Aldrich (St. Louis, MO, USA). Nitrocellulose (NC) membrane was procured from BioRad (Chennai, India), while TMB liquid substrate for HRP and a RevertAid First Strand cDNA synthesis kit was used from Thermo Fisher Scientific (Waltham, MA, USA). Primers for carbonic anhydrase IX (*CAIX*), *BAX, BAD, BCL2, VEGF*, and *MMP2* were purchased from Integrated DNA Technologies (Coralville, IA, USA), RNAisoPlus (Trizol) and qPCR SYBR green master mix both were from TaKaRa (Shiga, Japan), and the bicinchoninic acid (BCA) reagent was purchased from Thermo Fisher Scientific (USA). Antibodies against CAIX (A1658) and β actin (E-AB-20034) were acquired from ABclonal (Woburn, MA, USA), and their respective rabbit HRP-conjugated secondary antibodies were from Santa Cruz Biotechnology (Dallas, TX, USA). The chemicals lithium lactate, sodium pyruvate, and nicotinamide adenine dinucleotide (NAD) were purchased from SRL (Mumbai, India). The reference drug, Doxorubicin, used throughout the study, was purchased from Selleck Chemicals (Houston, TX, USA), and all of the in silico analyses were carried out using the Maestro, Schrödinger suite with the following specifications: Schrödinger Release 2022-3: Protein Preparation Wizard; Epik, Sitemap, Induced Fit Docking protocol; GLIDE module, Schrödinger, LLC, New York, NY, 2019, and GROMACS 2023 version.

### 5.2. General Procedures for the Preparation of C1 Isolated from the Seagrass Cymodocea serrulata, Source of Cell Lines and Routine Maintenance for Biological Assays

The *Cymodocea serrulata* collection, sample quantity, and appropriate permissions for sample collection as well as authentication, extraction and purification of the active compound can be found in our previous work in our laboratory [11], and no modifications have been made to the current study. Appropriate permission for sample collection has been obtained from the Central Salt and Marine Chemicals Research Institute (CSMCRI)-CSIR, Ramanathanpuram, Tamil Nadu, India-623519. The samples were sent to Dr. V. Veeragurunathan, and this information is accessible to the public for referencing purposes. IUCN lists *Cymodoceae serrulata* in the “Least Concern” category for Indian coastal waters. Column purification of the chloroform extract using hexane and ethyl acetate (90:10) as eluting solvents was performed to isolate C1, and the entire methodology, including column conditions, purification and spectral information, is detailed in the reference cited above. A yellowish–green semi-solid viscous compound (90 hexane: 10 ethyl acetate fraction Rf: 0.6; obtained as one among the 46 fractions eluted; yielded 140 mg of C1/500 g of dried seagrass biomass; compound yield: 0.028%) was shown to possess anticancer property. The compound was analysed using Fourier transform–infrared (FT-IR) spectroscopy, gas chromatography–mass spectrometry (GC-MS), electrospray ionization–mass spectroscopy (ESI-MS), ^1^H (proton) and ^13^C (carbon) nuclear magnetic spectroscopies (NMR). An exhaustive spectral analysis revealed that C1 is an ester of 3, 4-dihydroxy cinnamic acid and linoleic acid with glycerol with the IUPAC name [3-(E-3, 4-dihydroxycinnamaoyloxyl)-2-hydroxypropyl 9Z, 12Z-octadeca-9, 12-dienoate] and a molecular weight 516.67 Da. All the spectroscopic and spectrometric data are available at https://static-content.springer.com/esm/art%3A10.1038%2Fs41598-021-90845-9/MediaObjects/41598_2021_90845_MOESM1_ESM.pdf (accessed on 24 August 2025) [*kindly copy and paste the link on your browser*].

A stock solution of C1 was prepared using tissue culture grade dimethyl sulfoxide (DMSO) and stored at −20 °C until further use. A working solution of (1 mg/mL) was prepared from the stock and used for in vitro assays using lung adenocarcinoma cell line (A549) and the Vero cell line, a non-cancerous kidney cell line from African green monkey, which were acquired from the National Centre for Cell Sciences (NCCS), DBT, Pune, India. Both cell types (5 passages) were grown in a 25 cm^2^ cell culture-grade flask containing DMEM (Dulbecco’s Modified Eagle Medium) with an optimal pH of 7 and complemented with 10% FBS (fetal bovine serum). A combination of antibiotic and antimycotic solution [penicillin (10,000 units); streptomycin (10 mg); and amphotericin B (25 µg) in 0.9% saline] was added to the medium to avoid any microbial growth. Cell growth was achieved by maintaining the cells at 37 °C and 5% CO_2_ in an incubator, and Trypsin (10 X) was used for regular passaging.

The cytotoxic property of C1 was investigated by MTT (3-(4, 5-dimethylthiazol-2yl)-2, 5 diphenyl tetrazolium bromide) dye. To carry out this assay, approximately 1 × 10^3^ cells per well was planted in 96-well culture plates and allowed to grow until 80% confluence was attained. Affluent cells were induced with C1 (concentration 1–25 µM) for 24 h. At the end of the 24 h timeframe, the spent media was drained out, and 100 μL (5 mg/10 mL) of MTT dye solution was added and incubated at 37 °C for 4 h. MTT dye is taken up only by the live cells, and blue colored formazan crystals are formed by the action of active mitochondrial dehydrogenases, which can be solubilised using DMSO (100 µL). The colored solution was spectroscopically measured at 570 nm (EnSpire Multimode Plate reader, Model: 2300 [PerkinElmer, Shelton, CT, USA]). The cytotoxicity of the compound is directly proportional to cell mortality, which was calculated with the following formula: [OD of treated cells/OD of untreated cells] × 100. Doxorubicin (Doxo) was used as positive control throughout the investigation, and untreated controls were also maintained for comparison. Inhibitory Concentration_50_ (IC_50_) values were obtained for the compound and positive controls, which were used to plan for further assays.

### 5.3. Protocols for Cell Staining, DNA Fragmentation and Scratch Assay

The experiment was initiated by plating 1 × 10^5^ cells of A549 and Vero in a 6-well plate and incubating them at 37 °C with 5% CO_2_, and upon confluence to 80%, they were treated with 11.61 µM concentration of C1 for 24 h. Phosphate-buffered saline (PBS pH 6.8)-rinsed control and C1 and Doxo-treated cells were stained with 10 µL of AO/PI (from the stock: 10 µg of AO/PI in 1 mL PBS), and fluorescent images were captured using suitable filters (AO: Ex_500_/Em_526_ nm; PI: Ex_493_/Em_636_ nm). C1-infused cells were also treated with DAPI (0.5 µg/mL) and immobilised (fixed) using 4% paraformaldehyde for 10 min. After this, the cells were permeabilised for 10 min with 0.2% Triton-X100, and the images were captured (filters used: Ex_359_/Em_461_ nm). In both the cases, phenotypic and nuclear modifications were viewed under 10x objectives in an Eclipse-inverted fluorescent microscope (Nikon Ti E series, Japan). To understand the DNA-fragmenting properties of C1, confluent A549 cells were treated, and at the end of 24 h, cell pellets were lysed using the cell lysis buffer [(25 mM) EDTA and (200 mM) NaCl in Milli-Q water], 10 mg/mL proteinase K (Genei, Bengaluru) and 10% sodium dodecyl sulphate (SDS). For efficient lysis, the cells were incubated overnight at 37 °C. Chromosomal DNA was extracted using phenol/chloroform/isoamyl alcohol (25:24:1) and ice-cold absolute alcohol, and the extracted DNA was dissolved in TE buffer [1M Tris-HCl—500 mM EDTA, pH 8] and stored at −20 °C until further use. DNA samples were run in 1.5% agarose gel stained with (1 mg/mL) ethidium bromide. DNA fragmentation, if any, was viewed in a UV transilluminator (UVP Bioimaging Systems, Upland, CA), and the images were captured. Cell migration (scratch assay) was performed on 80% grown A549 cells by creating a scratch on the six-well plate on which it was grown, using 10 µL sterile micropipette tips and then treating with C1/Doxo for 24 h. The migration of cells was observed, and images were captured using an inverted microscope at 0 and 24 h and compared with untreated controls by measuring the migrated distance using the following formula: (Width of the gap at 0 h—Width of the gap at 24 h/Width of the gap at 0 h) × 100 using Image-Pro Plus software (version 6.3, Media Cybernetics, Rockville, MD, USA).

### 5.4. Flow Cytometry Experiments for Cell Cycle and Mitochondrial Membrane Potential (MMP) Analysis and RT-PCR Determinations of Certain mRNAs

Approximately 3 × 10^4^ cells were cultured in a 6-well plate and allowed to grow as mentioned previously. Upon confluence, they were treated with C1 in serum-free media for 24 h, and an untreated control was maintained for comparison. Both the cell groups were pelleted using ice-cold PBS (2000 rpm for 10 min) and stained with (50 µg/mL) propidium iodide suspended in a hypotonic solution [(0.3 µg/mL) Triton-X-100 in 0.1% sodium citrate and RNase (40 µg/mL)]. Cell sorting was completed using a MoFlo XDP Cell Sorter (Beckman Coulter Model: ML99030, CA) at 488 nm to observe the distribution of cells across different mitotic phases, and the results were inferred using the built-in software (Summit, version 4.3.02,) A portion of the cells were stained with a cationic fluorescent dye, Rhodamine 123, to study the disruption of MMP, if any. For this, the control and treated cell groups were incubated with Rhodamine 123 for 30 min in the dark at 37 °C and harvested with ice-cold PBS using the above-mentioned procedure. Stained cells were excited at 540 nm in a BD Accuri TM C6 Plus (BD Biosciences, New Jersey) flow cytometer, and the loss of Δψmit was calculated based on the fluorescent reduction between the control and treated cells population.

Relative changes in the mRNA expression levels of the pro- (*BAX* and *BAD*) and anti-(*BCL2*) apoptotic/cell survival [angiogenesis (*VEGF*), metastasis (*MMP2*) and carbonic anhydrase IX (*CAIX*)] genes in C1-treated and untreated cell types were quantified in real time. For this, RNAisoPlus reagent was used to extract the total RNA, and the purity was checked at 260/280 nm using a NanoDrop spectrophotometer (Nanodrop 1000, Thermo Scientific, USA). The complementary DNA was obtained by reverse transcribing 2 µg of the extracted RNA using the RevertAid First Strand cDNA synthesis kit. Primers for *MMP2* and *VEGF* were designed using Primer3 software (v. 0.4.0, 2017), and other gene-specific primers were used from the available literature. The master mix for qPCR was set up using 2 x SYBR green, 5 pmol of primers and cDNA to a final volume of 20 µl. The PCR scheme is as follows: 50 °C for 2 min followed by 40 cycles of 95 °C for 10 min, 95 °C for 15 s and 60 °C for 1 min. In a 7500 Fast RT PCR machine (ABI, Carlsbad, CA), fluorescence for SYBR green was measured using the following filter: Ex_497_/Em_520_. *GAPDH* was employed as a housekeeping gene for normalisation for all these experiments. A comparative threshold graph is supplied as shown in Figure S1 for reference. The relative quantification of gene expression was performed from the threshold value (Ct) obtained using Relative Manager Software (QM, version 1.2) and from the raw data; 2^−ΔΔCT^ was used to calculate the fold change as outlined by Schmittgen and Livak (2008) [34].

### 5.5. Molecular Docking

The 3D conformation of the human CAIX (PDB ID: 5DVX) with a resolution of 1.6 Å was acquired and processed utilising the Schrödinger Protein Preparation Wizard module (Schrödinger Release 2022-23: Protein Preparation Wizard), according to established protocols. They attributed precise charges, bond orders, and atom types to the protein structure. Moreover, water molecules more than 5 Å away from the heteroatom group were eliminated. Furthermore, it required the completion of missing side chains and loops using the Schrödinger Prime Module (Schrödinger Release 2022-23: Prime). The Glide Receptor, Grid Generation module, constructed a grid box surrounding the ligand, which was intricately linked to the protein’s active site. TRS, GOL, and CL components, excluding the Zn^2+^ ion, were removed from the protein. A gelatinase A-PEX inhibitor ligand molecule was retrieved from the PubChem database (Substance SID:404333222) [Kindly check the url: https://pubchem.ncbi.nlm.nih.gov/substance/404333222 (accessed on 24 August 2025) for compound submission at the NIH maintained PubChem database] for energy minimisation using the OPLS4 force field. This optimisation process uses the MacroModel tool in Maestro, utilising the Polac–Ribiere conjugate gradient (PRCG) technique. The ligand structure with the lowest energy structure was chosen for subsequent studies. LigPrep was utilised to prepare the ligand molecules (Schrödinger Release 2022-23: LigPrep), and the docking process involved standard precision (SP) docking mode and extreme precision (XP) docking mode employing glide docking (Schrödinger Release 2022-23: Glide) to bind the ligands with the protein [35], respectively.

### 5.6. Molecular Dynamic Simulation

Molecular dynamics (MD) simulations were employed to confirm the interactions, stability, and functionality of identified ligand structures within the crystal structure of the catalytic domain of human CAIX at a resolution of 1.6 Å (PDB ID: 5DVX). The GROMACS 2023 software package [36] and the CHARMM36 all-atom force field were employed to execute 100 ns MD simulations of specific docked complexes [37]. The topology and parameter files for the ligands were generated utilising the CHARMM General Force Field (CGenFF) tool [38]. Subsequently, the docked complexes were positioned within an orthorhombic container and hydrated using the four-point TIP4P water model [39], ensuring a minimum distance of 1 nm between the docked ligand and protein system. Additionally, to replicate physiological conditions accurately, a 0.15 M NaCl solution was introduced to maintain the ionic strength of the solvent in the system. Periodic boundary conditions (PBCs) were consistently applied throughout the MD simulation. The energy minimisation procedure employed the steepest descent algorithm, featuring a maximum step of 0.01 nm and a 1000 KJ/mol/nm tolerance. The Linear Constraint Solver (LINCS) approach was utilised to maintain bond length constraints, and electrostatic estimation was carried out employing the particle mesh Ewald (PME) approach. The system underwent equilibration using canonical ensembles NVT and was subsequently exposed to an isothermal–isobaric ensemble (NPT) for 100 ps after energy minimisation. The simulations were conducted under identical conditions of temperature and pressure (300 K, 1 atm). During the production run, trajectories were generated at a frequency of 2 fs and saved every 2 ps. The complete simulation persisted for a duration of 100 ns. The initial assessments involved various parameters, including root mean square deviation (RMSD), radius of gyration (Rg), root mean square fluctuations (RMSFs), potential binding energy, solvent-accessible surface area (SASA), hydrogen bond (H-bond), principal component analysis (PCA), and free energy landscape, which were measured using GROMACS analysis programs.

### 5.7. Correlation of CAIX Protein Expression with Extracellular pH (pHe) and Lactate Dehydrogenase (LDHe) Levels for the Compound-Treated Cells for Revalidation

An A549 cell population of 1 × 10^6^ was seeded in a T25 cm^2^ flask and allowed to grow overnight. Confluent cells were induced with C1 and Doxo and cells without treatment serve as controls. The total protein from all the groups was extracted by lysing the cells using RIPA buffer [with 1% protease inhibitor], incubating them in ice for 10 min, and then centrifuging them. Appropriate protein quantifications were made using BCA reagent, and after that, approximately 20 µg of protein was resolved in a 10% SDS-PAGE and transferred to NC membranes. The transferred membrane was incubated overnight at 4 °C with primary antibodies for CAIX (1:1000) and β actin [endogenous control] (1:500). After overnight incubation, the membranes were washed and incubated with anti-rabbit HRP-conjugated secondary antibodies for 1 h at room temperature. TMB liquid substrates were used to develop the membrane post-secondary anti-antibody incubation to visualise the protein bands.

Extracellular pH and LDHe measurements were also made. A549 cells were seeded at a density of 3 × 10^4^ cells in 6-well cell-culture grade plates to reach 80% confluence. Confluent cells were divided into six groups: (1) Normoxic control, (1a) Cells treated with C1 (11.61 µM), (1b) Cells treated with Doxo (13.7 µM), (2) Hypoxia induction by adding 200 µM of cobalt chloride for 72 h (kept as hypoxic control), and (2a) Cells treated with C1 and 2b. The cells were treated with Doxo, and the treatment period was 24 h. Spent media were collected from plates of all the groups and immediately checked for pHe using an electrode-based pH meter (Systronics). The relative difference in the extracellular pHe (ΔpHe) was measured at 0 and 24 h of treatment in both groups. Simultaneously, the determination of LDHe levels was completed by mixing 0.1 mL volume of the media with 1 mL of buffered substrate (lithium lactate dissolved in glycine buffer) along with 0.2 mL of nicotine adenine diphosphate (NAD) and incubating at 37 °C for 15 min. To this, 1 mL of dinitro phenyl hydrazine (DNPH) was added, after which it was incubated for 15 min. After this, 1 mL NaOH (0.4 N) was added and the color (yellow) was read at 440 nm using sodium pyruvate as a reference standard. The LDHe concentration (µg) in 1 mL of the media was calculated using the standard formula: [OD of test/OD of reference standard] × the reference concentration corresponding to that exact OD.

### 5.8. Statistical Analysis

Biological assays were performed as triplicates throughout the study, and individual data points were expressed as the arithmetic mean ± standard error from three independent experimental outputs. The data were statistically quantified using Student’s test, and any probability values (*p*-value) less than 0.05 were considered significant. Software built-in instruments were up to date for the calculations made automatically (scratch assay, for cell cycle and MMP shifts [Summit, version 4.3.02]. The data were processed and analysed in FCS format, which is the standard file format for flow cytometry data. Approximately 15,000 events were acquired to infer the results, and all the FACS results are depicted as density plots. Based on the DNA content histogram, gates were drawn around the G1, S, and G2/M peaks to quantify cells in each phase. For qPCR analyses, Primer3 software v. 0.4.0, 2017 was used to customize parameters such as the desired primer length, optimal melting temperature, and GC content to avoid primer dimers and hairpins. We used the software in batch mode to design primers for multiple targets simultaneously.

## 6. Patents

Details of a patent on the separation of anticancer compounds from the seagrass *Cymodocea serrulata* that were published in the *Indian Gazette* are provided as Supplementary Material for the editor and reviewers. In addition to being submitted as extra information, the ^1^H and ^13^C NMR assignments for C1 (CDCl, 400 MHz) are reported to the NIH-maintained PubChem database [Compound Identification Number (CID): 145864801].

## Figures and Tables

**Table 1:** Molecular docking of human carbonic anhydrase IX (CAIX) with gelatinase A-PEX inhibitor complex: binding score, hydrogen bond formation, and interactive residues using Schrödinger Glide.

Protein with Ligand Complex	Docking_Score	Glide_XP_GScore	No. of Hydrogen Bonds	Interactive Residues
CAIX–C1	−7.5546	−7.5584	3	GLU242, ALA392

**Table 2:** Brief comparison of certain properties of C1 and Doxorubicin.

Parameters	C1	Doxorubicin
IC_50_ [µM]	13.7	11.61
% Cell migration	15.49	0.58
pHe differences-Untreated and treated groups of normoxia/hypoxia	0.3/0.6	0.4/1
LDHe levels [µg/mL] between untreated and treated groups	273 ± 1.9	274 ± 1.3

## Data Availability

Available upon request to the corresponding author.

## Supplementary Materials

The following supporting information can be downloaded at: https://www.mdpi.com/article/10.3390/ph19010132/s1, Figure S1: Real time quantification of DNA captured for the genes *BAX, BAD* and *BCL2* (A) and *MMP2* and *VEGF* (B) and Carbonic Anhydrase IX (*CAIX*) (C) induced with C1.
