# Supplementary material for: A Marine Anticancer Cinnamyloxyl Derivative with Unique Binding Sites at Carbonic Anhydrase IX (CAIX) Inhibits Adenocarcinomic A549 Cells
Source: Pharmaceuticals (Basel). 2026 Jan 12;19(1):132. doi: 10.3390/ph19010132 (PMC12845052; doi:10.3390/ph19010132)
Supplement: Supplementary file 1 [file pharmaceuticals-19-00132-s001.zip › pharmaceuticals-3795507-supplementary.pdf]

Supplementary figures

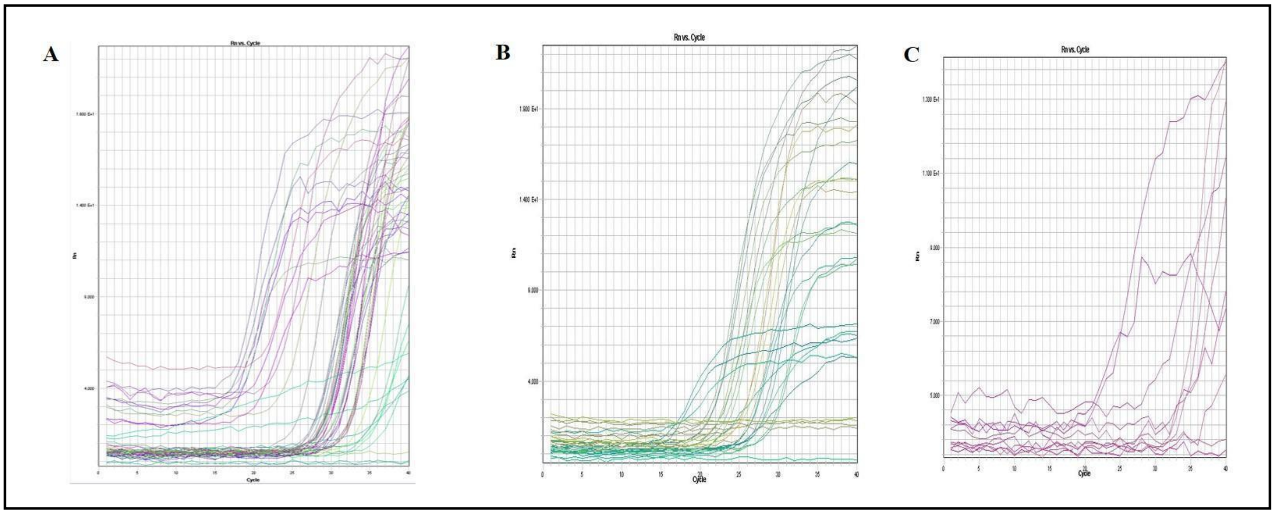

Figure S1. Real time quantification of DNA captured for the genes *BAX, BAD* and *BCL2* (A) and *MMP2* and *VEGF* (B) and Carbonic Anhydrase IX (*CAIX*) (C) when induced with C1
